# Concomitant chronic venous insufficiency in patients with peripheral artery disease: insights from MR angiography

**DOI:** 10.1007/s00330-020-06696-x

**Published:** 2020-02-25

**Authors:** Felix Ammermann, Felix G. Meinel, Ebba Beller, Anke Busse, Felix Streckenbach, Christine Teichert, Malte Weinrich, Andreas Neumann, Marc-André Weber, Thomas Heller

**Affiliations:** 1 Institute of Diagnostic and Interventional Radiology, Paediatric Radiology and Neuroradiology, University Medical Centre Rostock, Ernst-Heydemann-Str. 6, 18057 Rostock, Germany; 2 Department of General, Thoracic, Vascular and Transplantation Surgery, University Medical Centre Rostock, Rostock, Germany

**Keywords:** Magnetic resonance angiography, Venous insufficiency, Peripheral artery disease

## Abstract

**Objectives:**

The clinical presentation of peripheral artery disease (PAD) and chronic venous insufficiency (CVI) can overlap and the conditions may co-exist. The purpose of our study was to investigate the prevalence and clinical significance of concomitant CVI in patients with PAD examined with run-off MR angiography (MRA).

**Methods:**

We analysed 180 patients (median age 69 years, range 27 to 91) with known or suspected PAD who underwent MRA at our institution between 2012 and 2018. MRA datasets were re-evaluated for manifestations of CVI. Electronic charts were reviewed to analyse whether diagnosis of CVI was documented and to determine Fontaine stage of PAD.

**Results:**

Evidence of possible CVI on MRA was found in 38 (21%) patients. Only seven (18%) of these patients had a documented diagnosis of CVI. Patients with co-existing PAD and CVI were more likely obese (median BMI 29.7 vs. 26.3 kg/m^2^, *p* = 0.001) and diabetic (55 vs. 35%, *p* = 0.039) than patients without CVI. The frequency of concomitant CVI manifestations decreased from distal to proximal with the lower leg affected in all 38 patients and the thigh in 17 patients (45%). Patients with co-existing PAD and CVI were more likely to have a clinical diagnosis of stage IV PAD than patients without co-existing CVI (57% vs. 34%, relative risk 1.68, *p* = 0.018).

**Conclusions:**

Signs of possible concomitant CVI can be seen in approximately one-fifth of patients with known or suspected PAD examined with run-off MRA. If present, these findings should be reported since CVI may mimic or contribute to symptoms attributed to PAD.

**Key Points:**

*• In total, 21% of patients with PAD patients examined with MR angiography show signs of possible co-existing CVI.*

*• Patients with co-existing CVI were 1.7-fold more likely to have a clinical diagnosis of stage IV PAD.*

*• Our data also showed that co-existing chronic venous insufficiency is under-diagnosed in patients with PAD.*

## Introduction

Signs and symptoms of peripheral artery disease (PAD) and chronic venous insufficiency (CVI) can overlap and the conditions may be challenging to differentiate clinically in patients with leg pain or ulcers. Both conditions are extremely prevalent and share common risk factors, and therefore frequently co-exist. Recognising the potential co-existence of CVI and PAD is clinically important since these patients may require both arterial revascularisation and treatment of CVI to improve claudication symptoms and allow skin ulcers to heal.

CVI is a common vascular disease of great medical and socioeconomic impact (1–2.5% of health care budgets in developed countries), affecting a large part of the population worldwide and significantly decreasing the quality of life of affected patients [1]. According to a representative sample of adults of all ages, around 23% of the adult population have varicose veins and 17% have CVI of the lower leg with symptoms varying from oedema to ulceration [2]. PAD is similarly frequent affecting 3–10% of the population. The prevalence of PAD increases with age and the number of vascular risk factors reaches up to 15–20% above the age of 70 years. Lower extremity PAD affects approximately 10% of the American population, with 30 to 40% of these patients presenting with claudication symptoms [3–9].

There is scarce data on the prevalence of co-existing PAD and CVI [10, 11]. Most published investigations on this topic focused on patients with leg ulcerations and found that mixed (arterial and venous) aetiology is found in 10–18% of ulcer patients [10, 12–15]. One study performed in a cohort of patients with CVI found that 17% of patients with CVI also have co-existing PAD [10]. A higher risk for PAD in CVI patients has also been observed in population-based studies [11, 15–17].

Run-off MR angiography of the pelvic and lower extremity arteries is a commonly performed diagnostic test in patients with PAD. Although focused on the arterial vasculature, MR angiography as a cross-sectional imaging test also visualises changes in the venous system. Therefore, the purpose of our study was to investigate the prevalence of concomitant CVI in patients with known or suspected PAD examined with run-off MR angiography (MRA).

## Material and methods

### Study design and ethical approval

The investigation was designed as a retrospective, single-centre cohort study. We included (1) adult patients who (2) underwent a contrast-enhanced run-off MRA at our institution between January 2012 and April 2018 due to (3) known or suspected PAD. We excluded (1) patients with other indications for MRA (2) incomplete examinations, (3) examinations with a modified protocol, (4) missing images or clinical information and (5) repeat investigations of identical patients. The study was approved by the institutional review board with waiver of informed consent.

### Patient selection

We identified eligible patients through a retrospective search of our radiology information system (Centricity 5.0, GE Healthcare). All consecutive patients meeting all inclusion criteria and none of the exclusion criteria were included in the analysis. Patients referred to MR angiography for suspected PAD (*n* = 15) were included into the analysis even if MR ruled out PAD (*n* = 4).

### MRA technique

MRA examinations were performed on a 3-T MRI scanner (Magnetom Verio, Siemens Healthineers). The protocol consisted of (1) localisers, including three imaging stacks oriented in the transverse, sagittal and coronal planes at 4 levels (the abdomen, pelvis, upper legs/knee, lower legs); (2) a pre-contrast T1-weighted 3D MRA sequence acquired in coronal orientation also at four levels; (3) a time-resolved angiography with interleaved stochastic trajectories (TWIST) of the lower legs; and (4) a post-contrast T1-weighted 3D MRA sequence also acquired in coronal orientation at 4 stations. For the time-resolved angiography, we used a fixed dose of 4 mL gadobutrol (Gadovist®, Bayer Vital GmbH), followed by 0.2 mmol/kg body weight for the MR angiography. Flow rate was 2 mL/s in all patients. All datasets including localisers, pre-contrast images and subtracted and non-subtracted post-contrast images were archived in our PACS (IMPAX 6.5.3, Agfa HealthCare).

### Image analysis for the presence or absence of CVI

MRA datasets of all 180 patients were re-evaluated by two experienced readers in consensus (one radiology fellow, one board-certified radiologists with sub-specialisation in cardiovascular imaging). Readers viewed localisers, pre-contrast, dynamic MRA and non-subtracted post-contrast MRA images to identify signs of chronic venous insufficiency. Thin-section images of pre-contrast and non-subtracted post-contrast MRA images were viewed in 3D multiplanar reformats using a 3D module within our PACS. The presence of superficial varicose veins was used as the central radiological criteria for diagnosing CVI on MRA.

### Detailed image analysis in patients with radiological signs of CVI

In the subgroup of patients with signs of chronic venous insufficiency, the same readers performed a more detailed image analysis. For each leg, the prevalence of superficial varicose veins was recorded for several anatomical levels: the groin, thigh, knee, lower leg, ankle. We further recorded whether the tributaries of the great saphenous vein and/or the small saphenous vein were affected and whether skin changes potentially caused by CVI (oedema and/or ulcers) could be seen on MRA.

### Analysis of clinical data

Review of electronic patient charts was performed to record cardiovascular risk factors and Fontaine stage of PAD at the time of the MRA examination. For patients with signs of CVI at MRA, we further recorded whether the diagnosis of CVI was known at the time of the MRA examination.

### Statistical analysis

Statistical analysis was performed with GraphPad Prism 5. Descriptive statistics were used to analyse patient characteristics as well as the frequency and location of findings suggestive of CVI in the study cohort. Continuous data (age and body weight) were tested for normal distribution using the Kolmogorov–Smirnov test and found to be not normally distributed. Thus, continuous data were presented as median and range and compared using the nonparametric Mann–Whitney test. Categorical data were displayed as absolute frequencies and proportions. Fisher’s exact test was used to compare the frequency distribution of binary data between groups. Multiple logistic regression analysis was performed to identify independent predictors of stage IV peripheral artery disease. The following parameters were entered into the model as potential predictors: age, gender, BMI, smoking, diabetes, arterial hypertension, dyslipidaemia, renal insufficiency and signs of CVI on MRA. Odds ratios as well as corresponding 95% confidence intervals and *p* values were calculated. *P* values of < 0.05 were regarded as statistically significant.

## Results

### Patient characteristics

Patient characteristics are summarised in Table 1. All 180 patients in the cohort had known (*n* = 165, 92%) or suspected (*n* = 15; 8%) PAD. The majority was male (77%). The median age was 69 years, with a range from 27 to 91 years. The prevalence of cardiovascular risk factors was high with arterial hypertension (57%), diabetes (39%) and dyslipidaemia (39%) being the most frequent. Substantial cardiovascular comorbidity was present with a documented history of coronary artery disease in 24% of patients and cerebrovascular disease in 8%. On MR angiography, evidence of PAD was seen in 176/180 patients (98%) including all 165 patients with known PAD and 11/15 patients with suspected PAD. Of the remaining 4 patients, one had acute embolic arterial occlusion and 3 had normal arterial findings.

**Table 1:** Characteristics of the study population

		All patients(*n* = 180)		CVI(*n* = 38)		No CVI(*n* = 142)		*p* value
		*n*	%*	*n*	%*	*n*	%*	
Females		42	23%	11	29%	31	22%	0.390
Age in years, median (range)		69 (27–91)		70 (29–91)		68 (27–88)		0.061
BMI (kg/m^2^), median (range)		27.1 (17.3–53.3)		29.7 (21.2–40.4)		26.3 (17.3–53.3)		*0.001*
Indication for MRA	Known PAD	165	92%	35	92%	130	92%	1.000
	Suspected PAD	15	8%	3	8%	12	8%	
Cardiovascular risk factors	Smoking	53	29%	7	18%	46	32%	0.111
	Diabetes	71	39%	21	55%	50	35%	*0.039*
	Arterial hypertension	103	57%	25	66%	78	55%	0.270
	Dyslipidaemia	70	39%	16	42%	54	38%	0.709
	Renal insufficiency	20	11%	4	11%	16	11%	1.000
Vascular comorbidities	Coronary artery disease	44	24%	11	29%	33	23%	0.525
	Cerebrovascular disease	14	8%	4	11%	10	7%	0.498

*P* values < 0.05 are italicised

*Unless stated otherwise

### Patients with signs of co-existing CVI on MRA

**Fig. 1:**
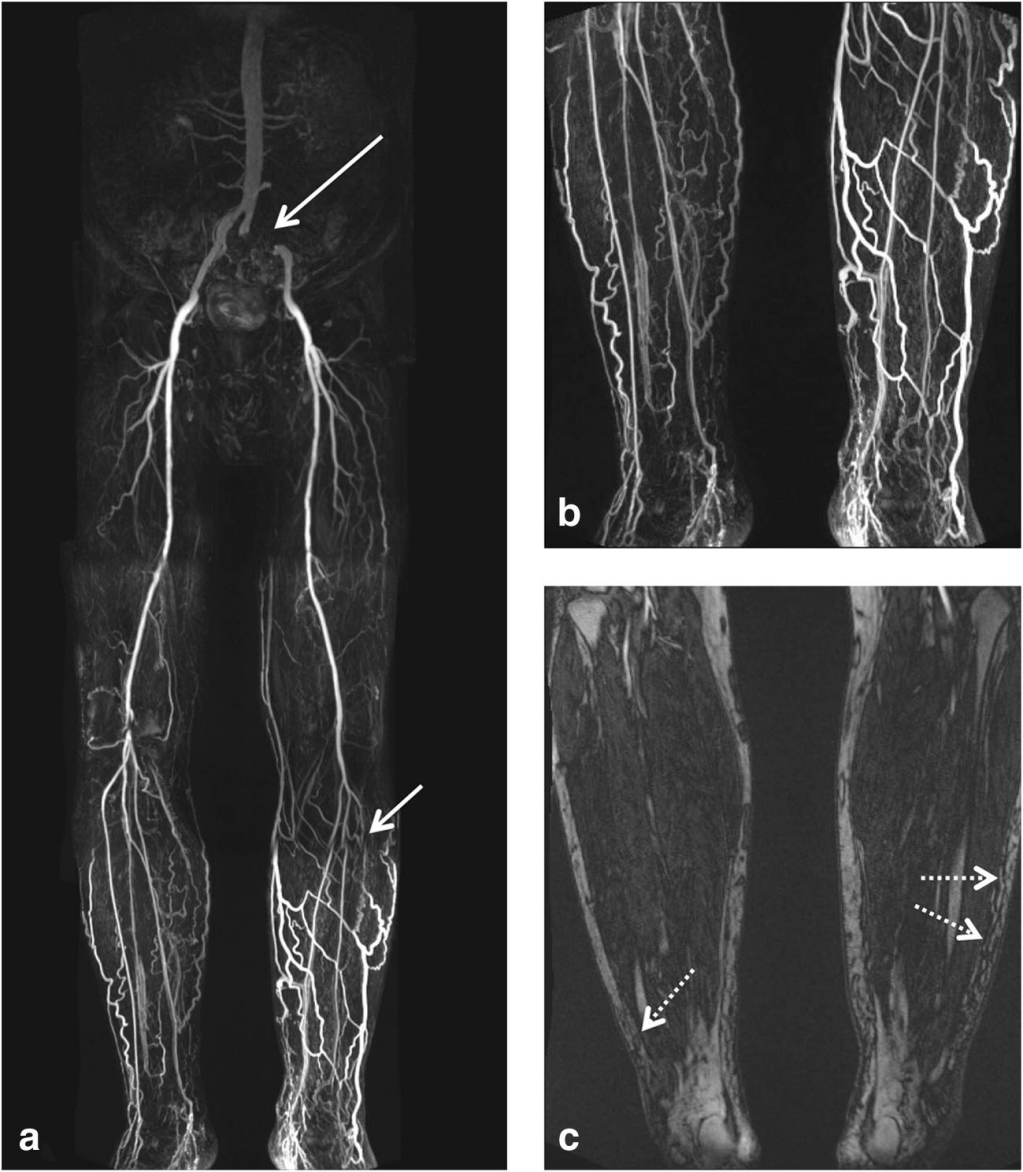
Run-off MR angiography in a 66-year-old male patient with known stage IV peripheral artery disease. Subtracted images post contrast show occlusion of the left common and internal iliac arteries (long arrow in **a**) and occlusion of the left anterior tibial artery (short arrow in **a**). Substantial varicosis of the superficial veins of both lower legs is also noted (magnified in **b**). T1-weighted pre-contrast image shows subcutaneous lower leg oedema (dotted arrows in **c**)

**Fig. 2:**
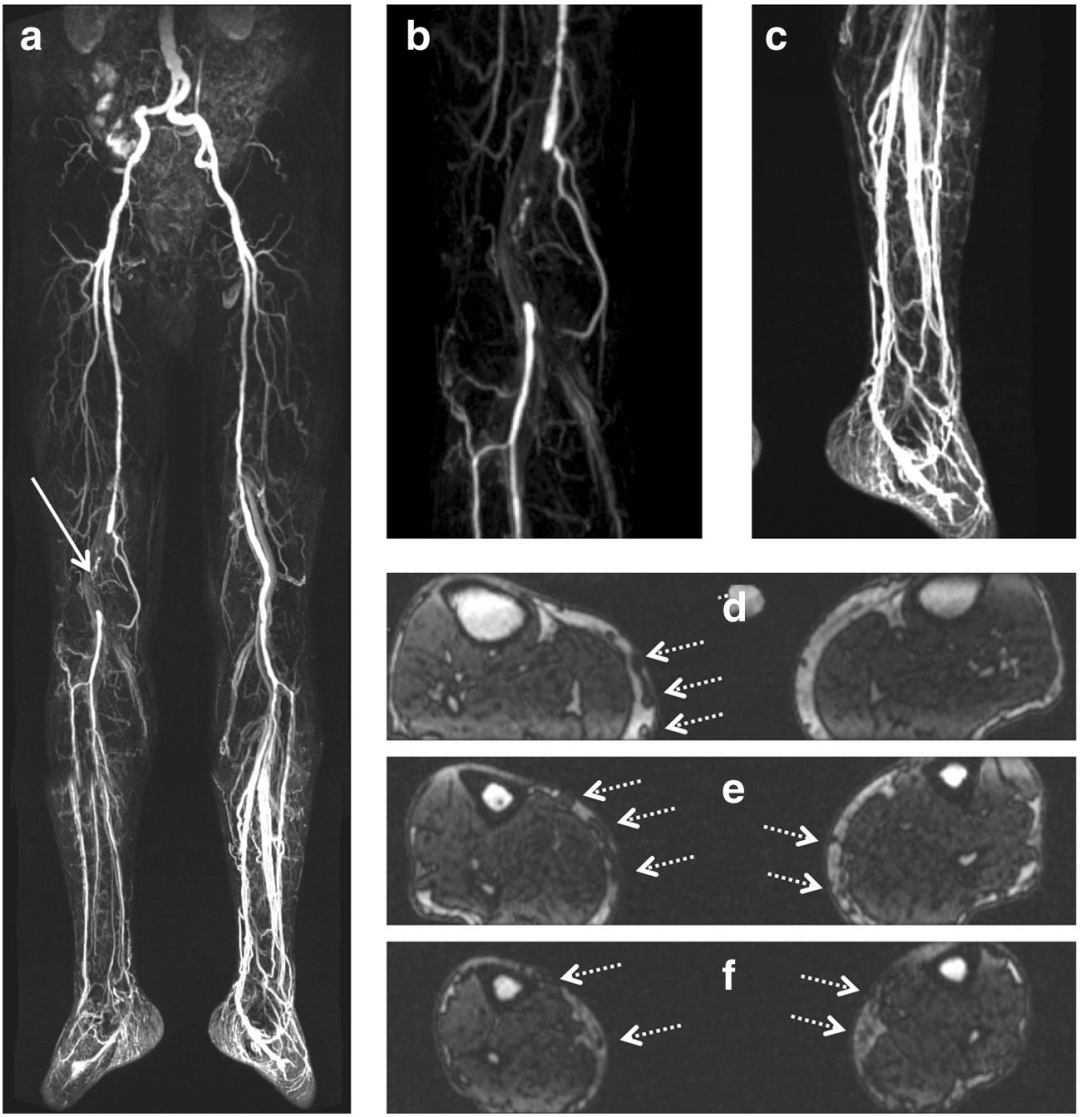
Run-off MR angiography in a 77-year-old male patient with known stage IIb peripheral artery disease. Subtracted images post contrast show occlusion of the right popliteal artery (arrow in **a**, magnified in **b**). There is substantial dilatation of the deep venous system particularly in the left lower leg (**a**, magnified in **c**). T1-weighted pre-contrast images show varicosis of the superficial veins of both lower legs medially (dotted arrows in **d–f**)

Signs of possible co-existing CVI on MRA were found in 21% of patients (*n* = 38, 27 men and 11 women; Figs. 1 and 2). In 7 (18%) of these 38 patients, CVI was documented as a diagnosis in the patient records. There was no difference in age or gender distribution between patients with and without signs of CVI on MRA (Table 1). Patients with co-existing CVI had a higher median body mass index than patients without signs of CVI (29.7 vs. 26.3 kg/m^2^, *p* = 0.001) and were more likely to be diabetic (55% vs. 35%, *p* = 0.039; Table 1).

### Distribution of varicosis and skin changes in patients with signs of co-existing CVI on MRA

Among the 38 patients with radiological evidence of CVI on MRA, CVI was bilateral in 28 patients (74%): left-sided in 7 patients (18%) and right-sided in 3 patients (8%, Table 2). All affected patients had varicose changes in the superficial veins on the lower legs. The frequency of manifestation decreased proximally with the level of the knee affected in 23 patients (61%), the thigh in 17 patients (45%) and the groin in 6 patients (16%). The great saphenous vein drainage system was involved in all patients; 8 patients (21%) had additional involvement of the small saphenous vein. Soft tissue oedema was seen in 24 patients (63%) and ulcers were seen in 20 patients (53%).

**Table 2:** Findings in patients with signs of CVI on MRA (*n* = 38)

		*n*	%
Affected legs	Right only	3	8
	Left only	7	18
	Bilateral	28	74
Varicose superficial veins	Groin	6	16
	Thigh	17	45
	Knee	23	61
	Lower leg	38	100
	Ankle	23	61
Skin changes	Oedema	24	63
	Ulcer	20	53

### Clinical significance

To determine the clinical significance of co-existing CVI in patients with PAD, we compared Fontaine stages of PAD between patients with and without signs of co-existing CVI (Table 3). Patients with co-existing CVI were more frequently in stage IV PAD (defined by ulcers or gangrene of the limb) than patients without co-existing CVI (57% vs. 34%, relative risk 1.68, *p* = 0.018). On multivariate logistic regression analysis (Table 4), co-existing CVI was the only significant independent predictor of stage IV PAD (odds ratio 3.139, confidence interval 1.318–7.760, *p* = 0.011).

**Table 3:** Clinical significance: analysis for 152 patients with known PAD and available Fontaine stage

	All patients (*n* = 152)		CVI (*n* = 35)		No CVI (*n* = 117)		*p* value
	*n*	%	*n*	%	*n*	%	
Fontaine stage IIa	4	3	2	6	2	2	0.227
Fontaine stage IIb	73	48	12	34	61	52	0.083
Fontaine stage III	15	10	1	3	14	12	0.193
Fontaine stage IV	60	39	20	57	40	34	*0.018*

*P* values < 0.05 are italicized

**Table 4:** Multiple logistic regression analysis for predictors of stage IV PAD

Variable	Odds ratio	95% confidence interval for odds ratio	*p* value
Age (per year)	1.010	0.974–1.047	0.600
Female gender	0.587	0.237–1.370	0.231
BMI (per kg/m^2^ unit)	0.976	0.895–1.061	0.569
Smoking	1.137	0.515–2.497	0.748
Diabetes	1.794	0.872–3.717	0.113
Hypertension	1.043	0.490–2.249	0.913
Dyslipidaemia	0.510	0.233–1.077	0.083
Renal insufficiency	2.280	0.798–6.558	0.121
Signs of CVI on MRA	3.139	1.318–7.760	*0.011*

*P* values < 0.05 are italicised

## Discussion

Our study investigated the prevalence, characteristics and clinical significance of co-existing CVI in patients with known or suspected PAD examined with run-off MRA. We found signs of co-existing CVI in 21% of patients. Patients with co-existing CVI were more likely to be obese and diabetic than patients without signs of CVI. Interestingly, we found that patients with co-existing PAD and CVI were 1.7-fold more likely to have a clinical diagnosis of stage IV PAD than patients without co-existing CVI.

All anatomic compartments, superficial and deep veins and perforators may be affected in CVI. CVI is caused either by impaired valve function of the superficial veins which causes retrograde blood flow and increased hydrostatic pressure or by failure of the valves in the perforator veins which allows blood to flow from deep veins backwards into the superficial system. The increased local pressure leads to venous dilatation and secondary failure of the superficial venous valves [18–21]. The changes of the venous anatomy such as ectasia of the deep veins and varicose changes of the superficial (subcutaneous) veins as well as secondary effects of CVI including ulcerations, oedema and skin thickening can readily be detected by cross-sectional imaging.

In clinical practice, cross-sectional imaging is not commonly used for the diagnosis of CVI with duplex ultrasound being the standard imaging tool [22, 23]. This may explain why radiologists do not focus on signs of CVI on cross-sectional imaging, especially when the clinical indication of MR angiography is known or suspected arterial disease. Previous studies have shown that CT and MR angiography have high accuracy for the evaluation of the lower extremity venous system [24–27] with the potential advantage of being better standardised and reproduceable than duplex ultrasound. Most prior studies on MR venography of the lower extremities focused on the detection of deep venous thrombosis or obstruction [28–30]. There is limited date on the use of MR for CVI and varicose veins [26, 31]. However, it should be noted that these previous studies used dedicated MRI protocols to evaluate the venous tree. In our investigation, we retrospectively assessed veins on MR angiography examinations tailored primarily to the evaluation of the arterial vasculature. The accuracy of this approach is likely limited compared with that of dedicated venous MRI protocols. In addition, skin changes are particularly difficult to evaluate from MRI at 3 T due to coil inhomogeneity. Our study design can identify patients with signs of possible CVI on MRI but cannot establish a definite diagnosis of CVI. Because the signs, symptoms and risk factors of PAD and CVI overlap, the conditions frequently co-exist [15] and can be difficult to differentiate clinically. Our cohort study suggests that co-existing CVI is greatly under-diagnosed in patients with PAD with only 18% of individuals with signs of CVI on MRA having a diagnosis of CVI documented in their records. Based on our results, we recommend looking for and reporting on signs of CVI if present on cross-sectional imaging performed in patients with PAD even though the focus of the examination is on arterial disease. In our patient cohort, we found a higher proportion of individuals who were obese or diabetic in the group of patients with co-existing PAD and CVI.

If signs of CVI are seen on cross-sectional imaging in patients with PAD, our recommendation would be to refer these patients for detailed venous evaluation by duplex ultrasound. Duplex ultrasound can directly visualise the direction of blood flow and thus demonstrate valve incompetency and venous reflux. Detailed venous evaluation by duplex ultrasound is also necessary to plan potential treatment for CVI. Recognising the potential co-existence of CVI and PAD is particularly relevant in patients with leg ulcers. Although some of the subgroups in each stage of PAD were relatively small, our study suggests that patients with co-existing PAD and CVI are more likely to have a clinical diagnosis of stage IV PAD (defined by ulcers or gangrene of the limb) than patients without co-existing CVI. The most likely explanation for this finding is that co-existing CVI potentiates the risk of ulcerations in patients with PAD (although the higher prevalence of diabetes in this subgroup could be a confounding factor). The clinical management of patients with mixed ulcers (caused by both arterial and venous pathology) can be challenging and may require both arterial revascularisation and treatment of CVI for ulcers to heal [12, 13, 24].

This study is not without limitations. This was a retrospective, single-centre study. Clinical stage of PAD by Fontaine classification was available for most but not for all patients. Since the MRA protocol and contrast timing were focused on the evaluation of the arterial vasculature, the protocol may not have been optimal for the detection of CVI manifestations. It is therefore possible that our results underestimate the true prevalence of CVI in patients with PAD. Due to the retrospective nature of this investigation, an external reference standard such as duplex ultrasound was not available to validate the diagnosis of CVI. Further, we lacked the necessary clinical data on skin changes and aetiology as well as functional data on venous reflux and/or obstruction to classify the stage of CVI using one of the established venous scoring systems such as the CEAP classification.

In conclusion, signs of possible concomitant CVI can be seen in approximately one-fifth of patients with known or suspected PAD examined with run-off MRA. If present, these findings should be reported since CVI may mimic or contribute to symptoms attributed to PAD.

## Abbreviations

CVI: Chronic venous insufficiency
MRA: Magnetic resonance angiography
PAD: Peripheral artery disease
TWIST: Time- resolved angiography with interleaved stochastic trajectories
